# Uveitis in Longstanding Axial Spondyloarthritis and Its Association with Biologic Therapy Initiation: Data from the REGISPON-3 Cohort

**DOI:** 10.3390/jcm14197128

**Published:** 2025-10-09

**Authors:** Ana María Sánchez-León, María Lourdes Ladehesa-Pineda, María Ángeles Puche-Larrubia, María Carmen Ábalos-Aguilera, Desirée Ruiz-Vilchez, Alejandro Escudero-Contreras, Eduardo Collantes-Estévez, Carlos M. Collantes-Sánchez, Clementina López-Medina, REGISPON-3 Study Group

**Affiliations:** 1 Ophthalmology Department, Reina Sofía University Hospital, 14004 Córdoba, Spain; anam_1989_5@hotmail.com; 2 Medical and Surgical Sciences Department, University of Córdoba, 14004 Córdoba, Spain; lourdesladehesapineda@gmail.com (M.L.L.-P.); mangeles.puche@gmail.com (M.Á.P.-L.); mc.abalos@outlook.com (M.C.Á.-A.); desriee.ruiz@imibic.org (D.R.-V.); alexcudero2@gmail.com (A.E.-C.); educollantes@yahoo.es (E.C.-E.); z82cosac@uco.es (C.M.C.-S.); 3 Maimonides Institute for Biomedical Research of Córdoba (IMIBIC), 14004 Córdoba, Spain; 4 Rheumatology Department, Reina Sofía University Hospital, 14004 Córdoba, Spain

**Keywords:** axial spondyloarthritis, acute anterior uveitis, biologic therapy, bDMARDs

## Abstract

**Objectives**: To assess the incidence rate of anterior acute uveitis (AAU) in patients with longstanding axial spondyloarthritis (axSpA); to evaluate demographic and clinical characteristics associated with AAU development; and to determine the influence of AAU on bDMARD initiation and retention in this population. **Methods**: This two-timepoint cohort study analysed data from patients enrolled in the Spanish SpA registry REGISPONSER (2004–2007), who were re-evaluated 17 years later in the REGISPON-3 follow-up study (2021–2023). Information on the date of first AAU episode and bDMARD initiation was collected. Kaplan–Meier and Cox proportional hazards models were used to assess AAU incidence, predictors, and its association with time to bDMARD initiation and treatment retention. **Results**: A total of 299 patients with longstanding axSpA were included, of whom 33.4% experienced at least one episode of AAU, corresponding to an incidence rate of 1.15 per 100 person-years. The cumulative probability of a first episode of AAU increased with disease duration. The relative risk for developing a second episode after the first, compared to the overall risk of any episode in the total population, was 1.85 (95% CI: 1.34–2.57). In multivariable cox analysis, female sex and baseline enthesitis were independently associated with a higher risk of AAU. AAU did not significantly affect the likelihood of subsequent bDMARD initiation, with similar cumulative treatment probabilities in patients with and without AAU. Among treated patients, adalimumab was more frequently prescribed in those with a history of AAU. bDMARD retention rates at two and five years were comparable regardless of AAU status, suggesting that AAU was not associated with long-term treatment persistence. **Conclusions**: In patients with longstanding axSpA, the incidence of AAU increased steadily over time. However, the presence of AAU did not significantly influence bDMARD initiation or long-term retention in routine clinical practice.

## 1. Introduction

Spondyloarthritis (SpA) is a group of inflammatory rheumatic diseases characterised by inflammation of the axial skeleton and/or peripheral joints, and by shared clinical and genetic features. Traditionally, SpA has been classified into subtypes based on the predominance of axial or peripheral symptoms and the presence of extra-musculoskeletal manifestations. These subtypes include ankylosing spondylitis (AS), psoriatic arthritis (PsA), SpA associated with inflammatory bowel disease (IBD), reactive arthritis (ReA), and juvenile SpA (Juv-SpA) [1]. In 2009, the Assessment of SpondyloArthritis International Society (ASAS) introduced new classification criteria for axial SpA (axSpA), followed by criteria for peripheral SpA (pSpA) in 2011 [2].

The most common extra-musculoskeletal manifestation of SpA is acute anterior uveitis (AAU), which affects approximately 21–33% of patients and is primarily associated with HLA-B27 and longer disease duration [3,4,5]. However, the prevalence of AAU varies by SpA subtype—being more frequent in axSpA than in PsA [6]—and by geographic region, with higher rates reported in North America and Europe compared to Asia and Latin America [7]. This manifestation has a substantial impact on the patient’s quality of life, work productivity, and treatment decision making, making it a crucial feature of axSpA itself [8].

AAU is an intraocular inflammation primarily involving the iris and ciliary body. It typically presents with inflammatory cells in the anterior chamber of the eye and is one of the leading causes of acquired blindness in the developed world. AAU is the most common form of uveitis in SpA, usually presenting as sudden onset, unilateral anterior uveitis. Up to 50% of patients who experience an episode of AAU will develop recurrences [9]. Notably, in approximately 10% of cases, AAU represents the first clinical manifestation of SpA [8], highlighting the critical role of ophthalmologists in the early identification of the disease.

Among the key factors associated with the development of AAU in axSpA is disease duration—patients with longer disease courses are more likely to have experienced at least one episode of AAU. Despite this, data from long-term follow-up studies in patients with longstanding axSpA are lacking, hampering a comprehensive understanding of the natural history of AAU.

Recent findings from the DEvenir des Spondyloarthrites Indifférenciées Récentes (DESIR) cohort have shown an AAU incidence rate of 1.29 per 100 person-years in patients with early axSpA. Over the first five years of follow-up, AAU was associated with dactylitis, biologic treatment, and active inflammation on Magnetic Resonance Imaging (MRI) of the sacroiliac joints [10]. Additionally, data from the COMOSPA study suggest that patients diagnosed in the biologic era (post-2000) had a lower probability of developing AAU, likely due to the impact of biologic therapies [11]. However, these short- to mid-term studies might not fully capture the cumulative incidence of AAU or its long-term influence on treatment strategies. Given the chronic capture of axSpA, long-term follow-up is required to understand whether AAU contributes to an earlier initiation of biologic disease-modifying antirheumatic drugs (bDMARDs) in patients with longstanding disease.

Based on these considerations, we conducted the present study with the following objectives: (a) to assess the incidence rate of AAU in patients with longstanding axSpA; (b) to evaluate the demographic and clinical characteristics associated with the development of AAU in this population; and (c) to determine the influence of AAU on bDMARD initiation and retention in patients with longstanding axSpA.

## 2. Materials and Methods

### 2.1. Study Design and Population

This two-timepoint cohort study analysed data from patients enrolled in the Spanish spondyloarthritis (SpA) registry REGISPONSER (year 2004–2007) [12], who were subsequently re-evaluated 17 years apart in the REGISPON-3 follow-up study (2021–2023) [13]. Data were collected at a single timepoint in each registry, supplemented with retrospective information from clinical records, which allowed longitudinal comparisons within the same patient cohort over a 17-year period. Baseline data from REGISPONSER and follow-up data from REGISPON-3 were merged into a unified database for analysis. Patients were eligible for inclusion in the REGISPON-3 study if they: (a) received a clinical diagnosis of SpA by a rheumatologist at the REGISPONSER baseline visit; (b) met the European Spondyloarthropathy Study Group (ESSG) classification criteria [14]; (c) completed both the REGISPONSER and REGISPON-3 study visits; (d) provided written informed consent and were able to complete standardised questionnaires.

For this specific analysis, only patients diagnosed with axSpA by the treating rheumatologist at the REGISPON-3 visit were included. All of them also fulfilled the ASAS classification criteria for axSpA [2].

### 2.2. Variables

Data were collected during both the REGISPONSER and REGISPON-3 visits using similar case report forms, allowing them to be merged into a unified database for analysis. During both visits, face-to-face consultations were conducted, in which patients underwent physical examination, interviews, and a review of their clinical records.

- Sociodemographic variables: age, sex, education, and smoking status were collected.
- Disease-related variables: disease duration (time since the onset of symptoms and the study visit), diagnosis delay (time between the onset of symptoms and the diagnosis), any episode of peripheral symptoms (i.e., arthritis, enthesitis and dactylitis), diagnosis of extra-musculoskeletal manifestations (i.e., AAU, IBD, or psoriasis), HLA-B27 status and c-reactive protein (mg/L) were collected.
- Patient Reported Outcomes (PROs): Disease activity was assessed using the Bath Ankylosing Spondylitis Disease Activity Index (BASDAI) [15] and the Ankylosing Spondylitis Disease Activity Score (ASDAS) [16]. Functional status was evaluated with the Bath Ankylosing Spondylitis Functional Index (BASFI) [17].
- Treatment information: Data were collected on the use of non-steroidal anti-inflammatory drugs (NSAIDs), conventional synthetic DMARDs (csDMARDs), biologic DMARDs (bDMARDs), and targeted synthetic DMARDs (tsDMARDs) across the patients’ full history since disease initiation. Information on treatment initiation and discontinuation dates, types of ts/bDMARDs prescribed, and treatment duration were also obtained from clinical records and prescription data.

### 2.3. Outcomes

Information on AAU was collected as a key extra-musculoskeletal outcome. Patients were asked whether they had ever experienced an episode of AAU confirmed by an ophthalmologist, and this was verified in the clinical records. If this information was not found, then the patient was not considered as having this feature. For those with a history of uveitis, the number of episodes was recorded, along with the dates of the first and most recent episodes. This allowed for the assessment of both the presence and recurrence of uveitis over time, as well as the assessment of the time to first onset of uveitis.

### 2.4. Statistical Analysis

Descriptive statistics were used to summarise patient characteristics. Categorical variables were reported as frequencies and percentages, while continuous variables were expressed as means with standard deviations (SDs) or medians with interquartile ranges (IQRs), as appropriate. Comparisons between patients with and without a history of AAU were performed using the chi-square test or Fisher’s exact test for categorical variables, and Student’s *t*-test or Mann–Whitney U test for continuous variables, depending on the distribution.

Cox proportional hazards regression models were applied to identify baseline factors associated with the development of AAU, with hazard ratios (HRs) and 95% confidence intervals (CIs) reported. Univariate analyses were first conducted, and variables with a *p*-value < 0.300 were included in the multivariable model.

Kaplan–Meier survival analysis was used to estimate the time to bDMARD initiation and treatment retention rates according to AAU status. Among patients treated with bDMARDs, the distribution of specific agents was compared between those with and without AAU.

A two-sided *p*-value < 0.05 was considered statistically significant. All analyses were performed using RStudio, version 1.4.1106.

The study was approved by the Ethics Committee of Reina Sofía University Hospital in Córdoba, Spain (approval code: 1713-N-20, dated 29 September 2020). All participants provided written informed consent.

## 3. Results

### 3.1. Prevalence and Incidence of AAU

A total of 411 patients were included in the two-timepoint REGISPON-3 dataset (i.e., patients evaluated at both the REGISPONSER and REGISPON-3 visits). Of these patients, 299 had a diagnosis of axSpA and were included in the present analysis.

Among them, 212 (70.9%) were male, with a mean age of 60.6 years (SD 10.2), a disease duration of 36.7 years (SD 10.4), and 90.3% were HLA-B27 positive at the re-evaluation REGISPON-3 visit (Table 1).

**Figure 1:**
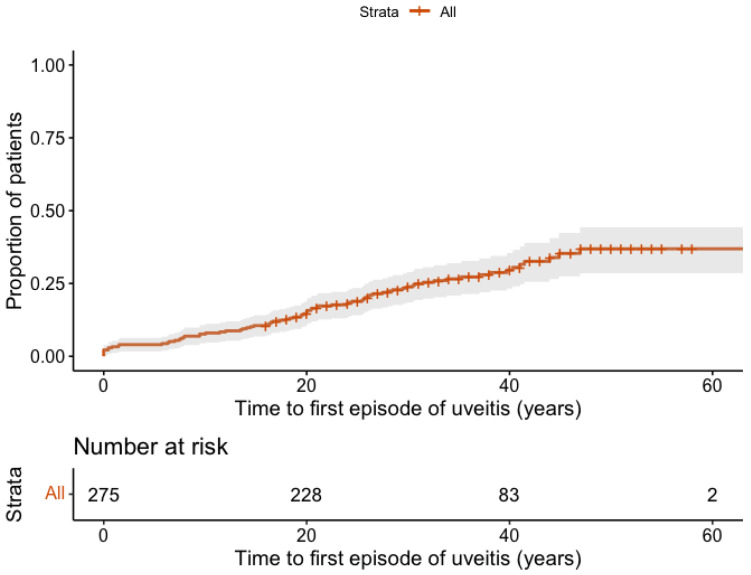
Kaplan–Meier curve showing the incidence of the first episode of AAU over time since axSpA symptom onset in the overall REGISPON-3 population. The *y*-axis represents the proportion of patients who developed a first episode of AAU. The *x*-axis represents the number of years since the onset of axSpA symptoms.

Within the study population, 100 out of 299 patients (33.4%) had experienced at least one episode of AAU by the end of the follow-up period, with a mean number of episodes of 4.0 (SD 4.6), and an incidence rate of 1.15 per 100 person-years. The Kaplan–Meier curve suggests a linear increase in the cumulative incidence of AAU over time, associated with disease duration (Figure 1). After 20 years of disease duration, the probability of having experienced a first episode of uveitis was 14.6% (95% CI 10.3–18.7), while after 40 years, this probability increased to 29.6% (95%CI 23.3–35.4).

**Figure 2:**
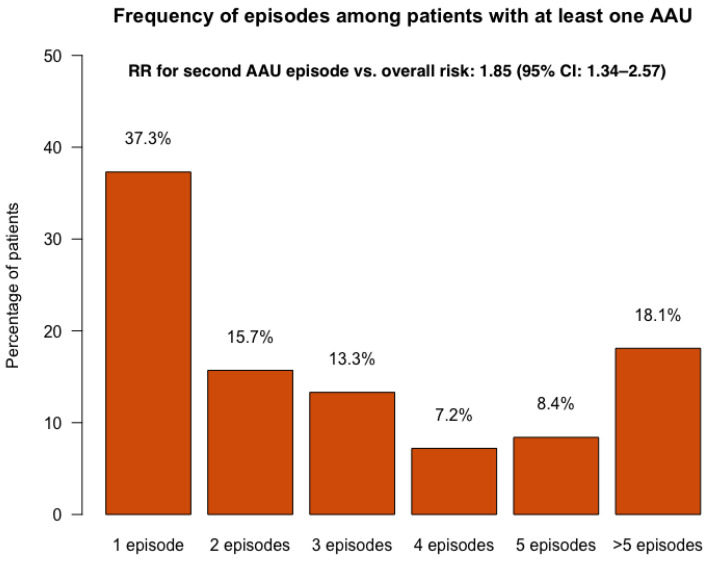
Distribution of first and recurrent acute anterior uveitis episodes in the REGISPON-3 population. AAU: acute anterior uveitis; CI: confidence interval; RR: relative risk.

Among patients who experienced at least one episode of AAU, 37.3% had only one episode, while 62.7% experienced more than one episode (Figure 2). This corresponds to a relative risk (RR) of 1.85 (95% CI: 1.34–2.57) for developing a second episode after the first, compared to the overall risk of any episode in the total population.

### 3.2. Baseline Predictive Factors of AAU

In the univariable Cox regression analysis (Table 2), none of the baseline variables reached statistical significance as predictors for the development of AAU, although male sex (HR 1.65, 95% CI 1.02–2.66, *p* = 0.074) and enthesitis (HR 1.43, 95% CI 0.91–2.26, *p* = 0.125) showed trends toward significance. Other clinical features, including HLA-B27 status, inflammatory back pain, synovitis, psoriasis, and family history of SpA, did not demonstrate statistically significant associations with AAU onset in the univariable model. Inflammatory markers (CRP, ASDAS) and baseline treatment with bDMARDs or csDMARDs were also not significantly associated.

In the multivariable Cox regression model (Table 2), which included variables with a *p*-value < 0.300 from the univariable analysis, female sex and the presence of enthesitis at baseline emerged as significant risk factors for AAU development (HR 1.65, 95% CI 1.02–2.66, *p* = 0.042, and HR 1.64, 95% CI 1.03–2.61, *p* = 0.038, respectively).

### 3.3. Influence of AAU on the bDMARD Initiation

Among patients with a history of AAU who had ever initiated a bDMARD (n = 54), 77.2% started the bDMARD after or at the time of the AAU episode, while 32.8% initiated the treatment prior to the development of AAU. For the remainder of the analysis, we focused on patients who initiated bDMARD therapy after the AAU episode.

**Figure 3:**
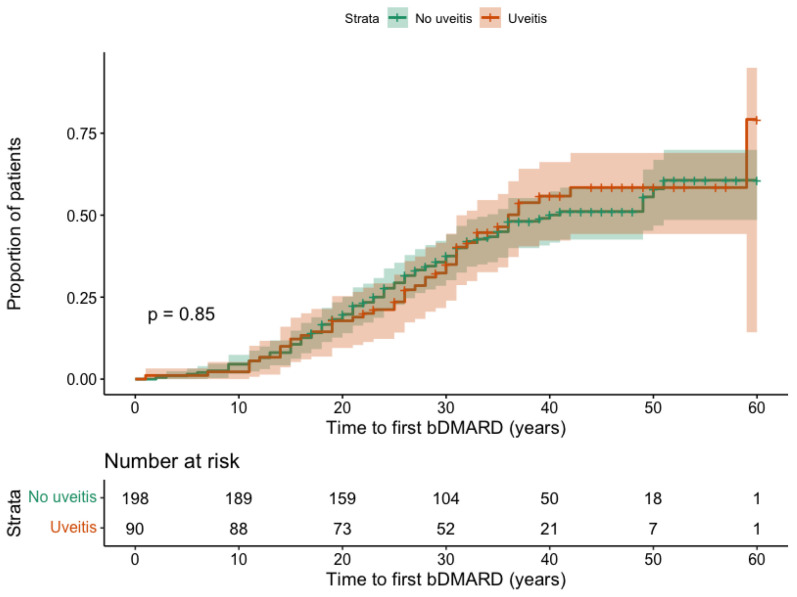
Time from symptom onset to first bDMARD initiation, stratified by history of acute anterior uveitis. The *y*-axis represents the proportion of patients who initiated a bDMARD. The *x*-axis represents the number of years since the onset of axSpA symptoms.

We then assessed whether a history of AAU was associated with a higher likelihood of subsequent bDMARD initiation (excluding those who started bDMARDs before the onset of AAU). Kaplan–Meier curves (Figure 3) showed that the cumulative probability of bDMARD initiation over time was similar between patients with and without AAU (*p* = 0.850). At 20 years following axSpA symptom onset, the probability of initiating bDMARDs was 17.8% (95% CI: 9.4 to 25.3) in patients with a history of AAU, compared to 19.7% (95% CI: 14.0 to 25.1) in those without AAU. After 40 years of disease duration, these probabilities increased to 55.8% (95% CI: 42.2 to 66.2) and 50.0% (95% CI: 41.6 to 57.3), respectively.

Among patients who had experienced AAU, the mean number of episodes was 3.5 (SD 2.5) in those who later initiated bDMARD therapy, compared to 4.4 (SD 5.4) in those who remained untreated, with no significant difference between the groups (*p* = 0.354).

In general, TNF inhibitors and IL-17 inhibitors were initiated at similar rates in patients with and without a history of AAU. However, adalimumab was the only specific agent significantly more prescribed in patients with a prior history of AAU compared to those without (71.7% vs. 41.9%, *p* < 0.001) (Table 3).

### 3.4. Influence of AAU on the bDMARD Retention Rate

**Figure 4:**
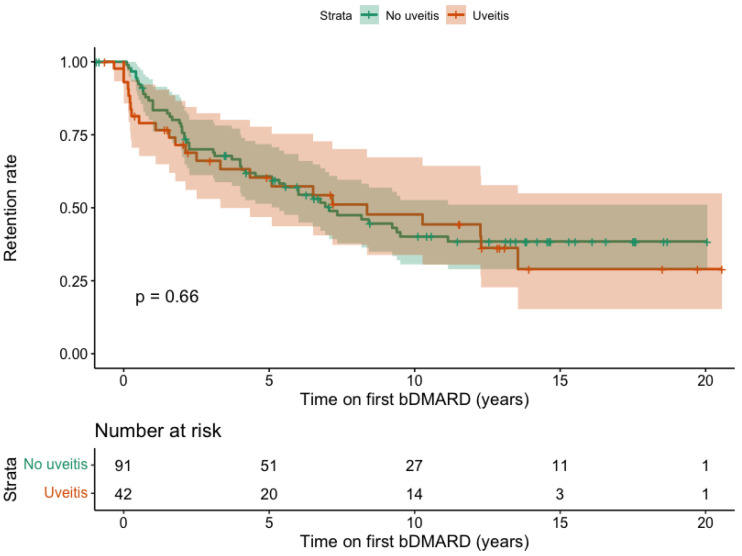
Retention rate of the first bDMARD, stratified by history of acute anterior uveitis. Legend: The *y*-axis represents the proportion of patients who discontinued the bDMARD. The *x*-axis represents the number of years since bDMARD initiation.

When analysing bDMARD retention, patients who experienced AAU prior to bDMARD initiation showed similar retention rates compared to those without a history of AAU (*p* = 0.660) (Figure 4). After two years of treatment, the probability of continuing bDMARD therapy was 71.5% (95% CI: 59.0 to 86.6) in patients with a history of AAU and 77.9% (95% CI: 69.8 to 86.9) in those without. At five years, these probabilities were 60.4% (95% CI: 46.8 to 77.8) and 60.7% (95% CI: 51.4 to 71.8), respectively.

## 4. Discussion

In this study of patients with longstanding axSpA from the REGISPON-3 cohort, we found that the incidence of new-onset AAU increases steadily over time. However, the presence of this extra-musculoskeletal manifestation does not significantly influence the initiation of a bDMARD, nor does it affect treatment retention.

Approximately one-third of patients had experienced at least one episode of AAU by the end of follow-up, with an overall incidence rate of 1.15 per 100 person-years. Notably, the cumulative incidence of AAU appeared to increase linearly over time, reinforcing the chronic and progressive nature of this complication. These findings are consistent with previous studies indicating that AAU is the most common extra-musculoskeletal manifestation in axSpA, and that its prevalence increases with longer disease duration [5,10,18]. In the DESIR cohort, a national French registry of patients with recent-onset axSpA, the incidence rate after five years of follow-up was 1.29 [0.84–1.74] per 100 patient-years, slightly higher than our findings, possibly due to differences in population characteristics [10].

Regarding baseline predictors, female sex and the presence of enthesitis were independently associated with a higher risk of developing AAU. Interestingly, we did not find a significant association with HLA-B27 positivity, a well-established risk factor for AAU [19]. This may be explained by the characteristics of our cohort: the REGISPON-3 cohort includes patients with longstanding disease, most of whom were diagnosed in the 1990s or early 2000s, when the concept of non-radiographic axSpA did not exist. All patients included had a clinical diagnosis of ankylosing spondylitis based on pelvic X-ray findings and the presence of the HLA-B27 antigen. Consequently, 90% of the patients were HLA-B27 positive, limiting the variability needed to detect a significant association.

The association between AAU and female sex has previously been considered controversial. Most studies have not identified sex differences in the incidence of AAU [1,5,11,20], whereas we found a statistically significant independent association with female sex. One possible explanation is that, historically, axSpA was considered a predominantly male disease. Therefore, the presence of an extra-musculoskeletal manifestation such as AAU in female patients may have increased diagnostic confidence among rheumatologists. As a result, the prevalence of AAU among women in our cohort may be inflated, as this clinical feature could have led to their inclusion in the registry.

Given that REGISPON-3 includes patients diagnosed before the widespread use of bDMARDs, we chose to analyse the association of AAU with the initiation of bDMARDs, rather than the effect of treatment on the development of AAU. In fact, in our cohort, only 32.8% of patients initiated biologic therapy before developing AAU. Therefore, we focused on those who started treatment after the onset of AAU to evaluate whether this manifestation influenced prescribing decisions. Contrary to expectations, a history of AAU did not significantly affect the timing of bDMARD initiation. Patients with and without AAU showed similar cumulative probabilities of starting biologic therapy over time. Furthermore, the number of AAU episodes did not differ significantly between treated and untreated groups. These findings suggest that, in real-world practice with longstanding axSpA patients, AAU may not be a key determinant in the decision to escalate to bDMARDs, although specific agents may be preferred in its presence. It should be noted, however, that the first bDMARD (adalimumab) was only approved for the indication of uveitis in 2016. Prior to this, other bDMARDs were used off-label in clinical practice for patients with axSpA and concomitant uveitis. The lack of approved treatment options for uveitis during the study period could therefore explain the non-significant prescription of bDMARDs in these patients.

Indeed, while the overall use of TNF and IL-17 inhibitors was comparable between patients with and without AAU, adalimumab (a TNF inhibitor with demonstrated efficacy in preventing AAU flares) was significantly more likely to be prescribed in patients with a history of AAU. This finding is expected, as adalimumab is one of the oldest available treatments for axSpA and the only bDMARD with a specific indication for patients with axSpA and concomitant AAU [21]. Moreover, recent meta-analyses have confirmed that the incidence of uveitis is lower with anti-TNF monoclonal antibodies compared to placebo [22]. IL-17 and JAK inhibitors have also shown a low incidence of AAU in clinical trials and meta-analyses, suggesting their potential effectiveness in reducing uveitis episodes [23,24,25,26,27]. However, no specific clinical trials have been conducted to validate these findings. In our study, the use of JAK inhibitors and bimekizumab was very limited, with no patients treated with the latter. This is likely due to the period of data collection (2021–2023), during which bimekizumab was not yet available and JAK inhibitors had only recently been approved.

Finally, bDMARD retention rates were not influenced by a history of AAU. After both two and five years of treatment, retention was similar between groups. This suggests that the presence of AAU does not negatively impact long-term adherence or the effectiveness of biologic therapy in routine clinical care. In fact, if a drug is effective in controlling uveitis, rheumatologists may be less inclined to switch therapies, given the limited number of agents with proven efficacy against this manifestation. Thus, treatment decisions may be more conservative in patients with AAU. To our knowledge, no prior observational studies have evaluated bDMARD retention in relation to the presence of uveitis, and future research is warranted to confirm these findings.

The main strengths of this study lie in its use of a real-world, longstanding cohort (REGISPON-3), which provides a unique opportunity to evaluate the natural history of AAU in axSpA patients diagnosed prior to the widespread use of bDMARDs. While many contemporary studies focus on early axSpA with the aim of understanding the initial stages of the disease, we believe it is equally important to study patients with longstanding disease to better understand its natural course and late-stage manifestations. Additionally, our analysis of bDMARD retention in relation to AAU status offers novel insights into real-world treatment behaviours, an area that has not been previously explored.

However, several limitations should be considered. The REGISPON-3 cohort predominantly includes HLA-B27 positive patients with radiographic axSpA diagnosed in the 1990s and early 2000s, which limits the generalisability of our findings to more recent or non-radiographic axSpA populations. Thus, these findings and conclusions, derived from this predominantly HLA-B27 positive radiographic cohort diagnosed before the biologic era, cannot be extended to nr-axSpA, to recent axSpA, or to contemporary cohorts. Furthermore, the low number of patients treated with newer agents such as JAK inhibitors or IL-17 blockers restricts our ability to draw firm conclusions regarding current prescription patterns in AAU. Additionally, treatment decisions and drug retention are influenced by multiple factors beyond AAU (such as efficacy, safety, comorbidities, and drug availability) which were not fully captured in this study. Finally, treatment switches were not evaluated, as it was not possible to determine whether the reason for these switches was the inefficacy in controlling uveitis.

## 5. Conclusions

In summary, in patients with longstanding axSpA, we observed a steady increase in the incidence of AAU over time, highlighting its chronic and progressive nature. Although AAU was common, it did not significantly influence the initiation or long-term retention of bDMARD therapy in clinical practice. Interestingly, female sex and the presence of enthesitis at baseline were independently associated with AAU, whereas HLA-B27 status was not. Further studies are warranted to validate these findings in more contemporary and diverse patient populations.

## Figures and Tables

**Table 1:** Characteristics of the patients included in the REGISPON-3 visit.

	Total = 299 N (%) or Mean (SD)
Sex (female), n (%)	87 (20.1%)
Age, mean (SD)	60.6 (10.2)
Ever smoker, n (%)	183 (61.2%)
Disease duration, mean (SD)	36.7 (10.4)
Diagnosis delay, mean (SD)	7.3 (8.3)
HLA-B27, n (%)	261 (90.3%)
Inflammatory back pain, n (%)	291 (97.9%)
Peripheral synovitis, n (%)	96 (32.7%)
Enthesitis, n (%)	75 (25.3%)
Dactylitis, n (%)	15 (5.1%)
Acute anterior uveitis, n (%)	100 (33.4%)
Psoriasis, n (%)	28 (9.6%)
IBD, n (%)	24 (8.0%)
Sacroiliitis, n (%)	268 (91.8%)
BASDAI (0–10), mean (SD)	3.6 (2.3)
CRP mg/L, mean (SD)	5.0 (9.7)
ASDAS, mean (SD)	2.5 (1.3)
BASFI (0–100), mean (SD)	36.8 (23.1)
mSASSS, mean (SD)	21.5 (22.5)
ts/bDMARD use ever, n (%)	147 (49.2%)
csDMARD use ever, n (%)	112 (37.5%)

ASDAS: Axial Spondyloarthritis Disease Activity Score; BASDAI: Bath Ankylosing Spondylitis Disease Activity Index; BASFI: Bath Ankylosing Spondylitis Function Index; CRP: c-reactive protein; csDMARD: conventional synthetic disease-modifying antirheumatic drug; IBD: inflammatory bowel disease; mSASSS: modified stoke ankylosing spondylitis spinal score; SD: standard deviation; ts/bDMARD: targeted synthetic or biological disease-modifying antirheumatic drug.

**Table 2:** Baseline predictive factors at the REGISPONSER visit for the development of AAU over time.

	Univariable Cox Regression HR (95%CI)	*p*-Value	Multivariable Cox Regression * HR (95% CI)	*p*-Value
Sex (female)	1.53 (0.96–2.43)	0.074	1.65 (1.02–2.66)	0.042
Diagnosis delay	0.99 (0.96–1.02)	0.350		
HLA-B27	1.34 (0.58–3.10)	0.487		
Axial pain	0.52 (0.13–2.14)	0.368		
Buttock pain	0.79 (0.49–1.29)	0.355		
Synovitis	0.76 (0.46–1.28)	0.304		
Enthesitis	1.43 (0.91–2.26)	0.125	1.64 (1.03–2.61)	0.038
Psoriasis	1.64 (0.66–4.06)	0.285	2.29 (0.90–5.82)	0.083
IBD	1.26 (0.39–4.01)	0.697		
Family history of SpA	1.22 (0.90–1.66)	0.201	1.18 (0.88–1.63)	0.249
Sacroiliitis	2.15 (0.53–8.77)	0.286	2.80 (0.68–11.5)	0.154
BASRI total	0.98 (0.92–1.04)	0.527		
CRP	0.99 (0.97–1.01)	0.439		
ASDAS	0.88 (0.70–1.12)	0.306		
bDMARD at baseline	1.26 (0.65–2.45)	0.500		
csDMARD at baseline	1.29 (0.75–2.22)	0.351		

* Final number of patients included in the multivariable Cox regression: n = 272. ASDAS: Axial Spondyloarthritis Disease Activity Score; BASRI: Bath Ankylosing Spondylitis Radiographic Index; CRP: c-reactive protein; csDMARD: conventional synthetic disease-modifying antirheumatic drug; IBD: inflammatory bowel disease; SpA: spondyloarthritis; bDMARD: biological disease-modifying antirheumatic drug.

**Table 3:** Type of ts/bDMARD ever prescribed after a first episode of AAU.

	Overall Population N = 139	Patients with a History of AAU N = 46	Patients Without a History of AAU N = 93	*p*-Value
TNF inhibitors	133 (95.7%)	45 (97.8%)	88 (94.6%)	0.664
- Adalimumab	72 (51.8%)	33 (71.7%)	39 (41.9%)	<0.001
- Etanercept	61 (43.9%)	18 (39.1%)	43 (46.3%)	0.427
- Infliximab	55 (39.6%)	19 (41.3%)	36 (38.7%)	0.769
- Certolizumab	9 (6.5%)	5 (10.9%)	4 (4.3%)	0.157
- Golimumab	29 (20.9%)	11 (23.9%)	18 (19.4%)	0.658
IL-17A inhibitors	21 (15.1%)	8 (17.4%)	13 (14.0%)	0.597
- Secukinumab	20 (14.4%)	8 (17.4%)	12 (12.9%)	0.478
- Ixekizumab	4 (2.9%)	1 (2.2%)	3 (3.2%)	1.000
- Bimekizumab	0	0	0	-
JAK inhibitors	2 (1.4%)	1 (2.2%)	1 (1.0%)	1.000
- Tofacitinib	0	0	0	-
- Upadacitinib	2 (1.4%)	1 (2.2%)	1 (1.0%)	1.000

AAU: acute anterior uveitis; IL-17A: Interleukin 17A; JAK: Janus kinase; TNF: Tumour Necrosis Factor.

## Data Availability

Data are available upon a reasonable request and after approval by the REGISPON-3 Scientific Committee.
